# Impact of Pulmonary Hypertension on Mortality after Surgery for Aortic Stenosis

**DOI:** 10.3390/medicina58091231

**Published:** 2022-09-06

**Authors:** Luminita Iliuta, Marius Rac-Albu, Madalina-Elena Rac-Albu, Andreea Andronesi

**Affiliations:** 1 Department of Medical Informatics and Biostatistics, University of Medicine and Pharmacy “Carol Davila”, 050474 Bucharest, Romania; 2 Cardioclass Clinic for Cardiovascular Disease, 031125 Bucharest, Romania; 3 Nephrology Department, University of Medicine and Pharmacy “Carol Davila”, 050474 Bucharest, Romania; 4 Nephrology Department, Fundeni Clinical Institute, 022328 Bucharest, Romania

**Keywords:** aortic stenosis, aortic valve, pulmonary hypertension, aortic valve replacement

## Abstract

*Background and Objectives:* The prognosis of patients with aortic stenosis (AS) adding pulmonary hypertension (PHT) is worse than in those with normal pulmonary artery pressure (PAP), and there are few results reported for the association between PHT and adverse outcomes of AS. We aimed to determine the predictive factors for the development of PHT in patients with surgical AS and to identify those factors that may predict the surgical prognosis. We aimed to identify the independent predictors for PHT regression at 2 years after surgery. Additionally, we tried to evaluate the involvement of PHT as an additional perioperative risk factor in patients with AS undergoing surgical aortic valve replacement (AVR). *Materials and Methods:* We carried out a two-year prospective study on 340 patients with AS undergoing surgical AVR. *Results:* The independent predictors for the occurrence of PHT in patients with surgical AS were: age > 75 years (RR = 6, *p* = 0.001), a restrictive left ventricle diastolic filling pattern (LVDFP) (RR = 9, *p* = 0.001) and associated moderate mitral regurgitation (MR) (RR = 9, *p* = 0.0001). The presence of severe PHT increased by 7.6 times the early postoperative risk of death, regardless of the presence of other parameters. The independent predictors for early postoperative mortality were: severe PHT, restrictive left ventricle diastolic pattern, age > 75 years, interventricular septum (IVS) thickness >18 mm and the presence of comorbidities. *Conclusions:* The presence of a severe PHT in patients with AS undergoing surgical AVR is associated with an early postoperative increased mortality rate. The mean PAP is a more reliable parameter for prognosis appreciation than the LV systolic function.

## 1. Introduction

Pulmonary hypertension (PHT) represents a condition of a decompensated disease state with exhausted compensatory mechanisms of the left ventricle in patients with severe aortic stenosis (AS) [1]. It means a heart failure state resulting from AS-related cardiac stress and/or injury [2], particularly in aging populations, and it is usually associated with increased morbidity and mortality. The prevalence of PHT in patients with AS varies significantly according to the severity of the stenosis, the presence of symptoms and the pressure cut-off used to define PHT, ranging from 15% to 30% [3]. Additionally, long-term prognosis in patients with AS with added PHT is worse than in those with normal PAP.

Pathophysiological adaptive mechanisms that lead to structural changes in the heart (left ventricular (LV) hypertrophy, left atrial (LA) dilatation and increased fibrosis in conjunction with impaired LV systolic function, concomitant MR and increased LV end-diastolic pressure) may explain PHT in AS [4].

Some studies failed to show an independent impact of preoperative pulmonary pressure on postoperative outcome. However, other series suggested that preoperative PHT is associated with increased mortality and decreased long-term survival, particularly in the case of severe PHT [5,6]. Additionally, previous studies have not evaluated the influence of PHT on postoperative course in these patients [7].

On the other hand, surgical options in elderly patients with multiple comorbidities may be limited and an important number of these high-risk surgical candidates may now be optimally managed using transcatheter aortic valve implantation (TAVI). That is why it is very important to evaluate accurately the surgical risk and to assess the impact of associated severe PHT in these patients for the ultimate decision to proceed with a conventional or transfemoral surgical procedure.

A few randomized clinical trials comparing the clinical outcomes of transfemoral-transcatheter aortic valve implantation (TF-TAVI) versus surgical AVR or transapical (TA)-TAVI are limited in summarizing the overall outcomes. These studies did not consider the importance of each risk factor, especially the presence of severe PHT.

There are also controversies about the outcomes for patients with PHT who undergo TAVI or surgical AVR. There is evidence that patients with PHT who underwent TAVI and surgical AVR had similar in-hospital mortality, but TAVI was associated with lower cardiac, respiratory and bleeding complications compared with surgical AVR [8].

The first objective of our study was to assess the predictive factors associated with the PHT development in patients with surgical AS. We tried also to define the independent predictors for immediate and long-term prognosis in these patients and their adjusted value. Finally, we tried to evaluate the PHT involvement as and additional perioperative risk factor in patients with AS who underwent surgical AVR.

## 2. Materials and Methods

The study was prospective and included 340 consecutive patients with AS who underwent surgical AVR in a single center (C. C. Iliescu Institute for cardiovascular diseases, Bucharest, Romania) in two consecutive years. There were not including patients with chronic atrial fibrillation, associated aortic valve insufficiency, acute myocardial infarction, or coronary lesions, associated mitral or tricuspid lesions. At the moment of the enrolment into study, all patients were in sinus rhythm. The study protocol was approved by the institute management and the informed consent form was signed by all patients.

Patients were evaluated clinically, by lab tests, electrocardiogram, and by echocardiography before surgery and postoperatively at 10 days; 1, 3, and 6 months; and at 1 and 2 years. The clinical assessment included: NYHA class for heart failure, hospitalization duration and quality of life (using the health-related quality of life questionnaire). To measure the quality of life, we used an adapted, abbreviated questionnaire, as presented in Appendix A.

Laboratory parameters included: the usual blood tests (platelet count, hemoglobin, hematocrit, aminotransferases, and lactate dehydrogenase), electrocardiogram (with the evaluation of rhythm and frequency) and echocardiographic measurements of the LV dimensions and performance (systolic and diastolic), left atrium dimensions and compliance.

Categorical data are presented as numbers and percentages and continuous data are given as mean ± standard deviation or median (interquartile range) as appropriate. Patients without and with medium or severe PHT were compared using chi-square tests, analysis of variance (ANOVA) or the Kruskal–Wallis test with appropriate post hoc tests. We also calculated Pearson or Spearman correlation coefficients for correlations of interest. Survival of patients without PHT and those with medium and severe PHT was compared using Kaplan–Meier curves and log-rank tests. The independent predictors of mortality were assessed using multivariate Cox regression analysis. We entered in the multivariate model the covariates associated with mortality in the univariate analysis (*p* < 0.1). Similarly, Kaplan–Meier plots were constructed to compare survival according to parameters of interest and a multivariate model was constructed to define the prognostic value of LVDFP and age. Statistical analysis calculates also relative risk, confidence intervals and correlation coefficient for the covariates of interest. A *p*-value < 0.05 was considered statistically significant. All analyses were performed using SPSS statistical package version 20.0.

The pulmonary artery pressure was evaluated using continuous Doppler examination and depending on its value, the patients were divided into three groups:

Group A—182 patients with normal PAP.
Group B—103 patients with mean PAP between 30 and 50 mmHg.
Group C—55 patients with mean PAP more than 50 mmHg [9].

All patients received treatment for heart failure according to current guidelines. The groups of patients were homogeneous in terms of treatment, the only exception being patients with PHT, who received increased doses of diuretics. Additionally, patients with PHT did not receive specific treatment (endothelin-receptor antagonists, phosphodiesterase type 5 inhibitors, soluble guanylate-cyclase stimulators, prostacyclin analogs or prostacyclin-receptor agonists). Most of the patients were male (65%), the mean age of the group was 62 years, the mean gradient between LV and aorta was 65 ± 12 mmHg, and the mean aortic valve area was 0.83 ± 0.22.

## 3. Results

Multivariate logistic regression analysis identified as independent predictors for PHT occurrence the following parameters:

- Age >75 years (RR = 6), *p* < 0.001;
- Restrictive LV diastolic filling pattern (RR = 9), *p* < 0.001;
- Associated moderate mitral regurgitation (RR = 9), *p* < 0.0001.

In this pattern, the prediction of the PHT occurrence was made with a sensitivity of 80% and a specificity of 85%.

**Figure 1:**
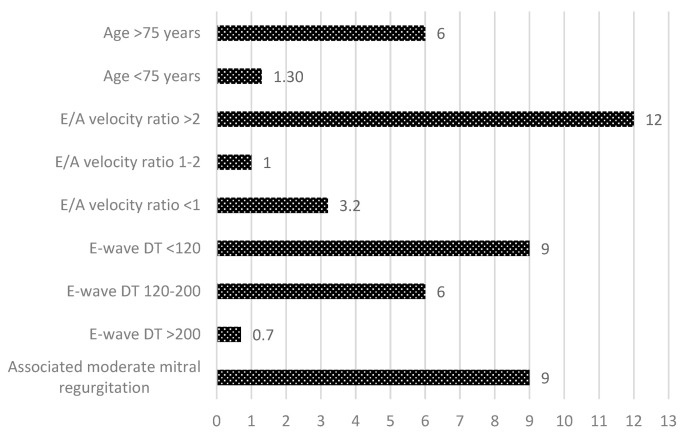
Relative risk for the occurrence of pulmonary hypertension in patients with aortic stenosis.

In Figure 1, the relative risks associated with the parameters that increase the risk of the PHT occurrence in patients with severe AS are presented. The risk of the presence of associated severe PHT to surgical AS increased about 6-fold in patients older than 75 years and about 9-fold by the presence of restrictive diastolic filling pattern (respectively, by E/A velocity ratio more than 2 by 12-fold and E wave deceleration time <120 ms by 9-fold). In addition, an associated second-degree mitral regurgitation increased the risk of PHT occurrence by 9-fold in patients with surgical AS.

The presence of severe PHT increased the early postoperative risk of death by 9.8-fold, regardless of the presence of other known parameters that increased mortality rate in these patients.

**Figure 2:**
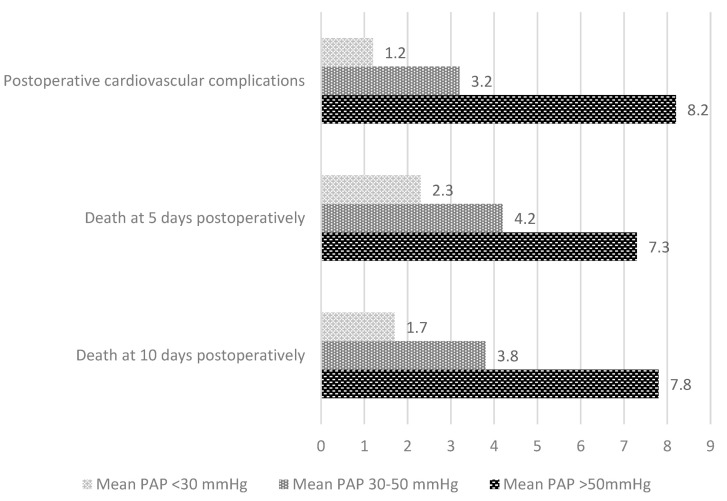
Relative risk for cardiovascular complications and early postoperative death depending on the presence of pulmonary hypertension.

In Figure 2, the relative risks associated with death at 5 and 10 days postoperatively are shown along with the appearance of postoperative cardiovascular complications, presented distinctly depending on the PAP value.

**Figure 3:**
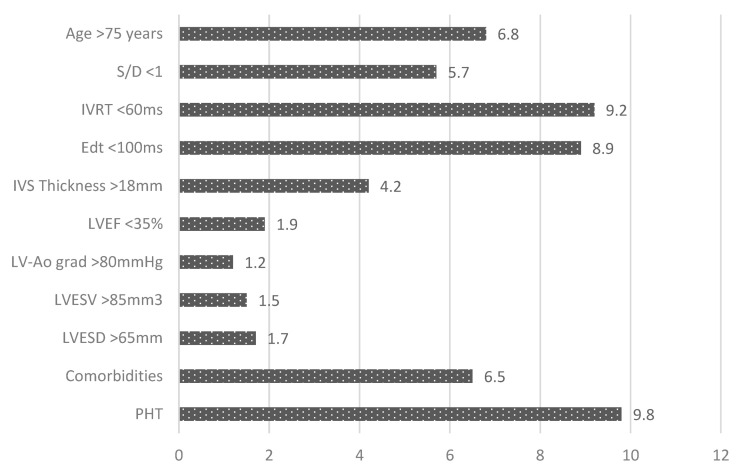
Early postoperative risk of death in patients with aortic stenosis.

Regression analysis identified as independent predictors for early postoperative mortality the presence in those patients of the following parameters (Figure 3):

- Mean PAP > 50 mmHg (RR = 9), *p* < 0.001;
- Restrictive LV diastolic filling pattern (RR = 9), *p* < 0.001;
- Age >75 years (RR = 6), *p* < 0.001;
- Interventricular septum thickness >18 mm;
- The presence of comorbidities (diabetes mellitus and chronic kidney disease) (RR = 9), *p* < 0.0001.

Parameters of the LV systolic performance (LVEF), severity parameters of aortic lesion (gradient, area) and LV dimensions were not correlated with the increased early postoperative mortality rate in these patients.

In Figure 3, the relative risks associated with early postoperative death for the parameters known to increase the mortality rates in surgical AS are presented.

Thus, the risk of early death postoperatively was increased about 10 times by the presence of PHT and by 9-fold by the presence of restrictive LV diastolic filling pattern (respectively, by the E wave deceleration time <100 ms by 8.9-fold and by the isovolumetric relaxation time <60 ms by 9.2-fold). In addition, a less than 1 ratio of systolic and diastolic waves in pulmonary veins increased the risk of early death postoperatively by 5.7-fold, patients’ age >75 years by 6.8-fold, and the eccentric left ventricular hypertrophy with interventricular septum thickness >18 mm by 4.2-fold. The early postoperative risk of death was increased as well by the associated comorbidities (diabetes mellitus, chronic obstructive pulmonary disease, and chronic kidney disease).

**Figure 4:**
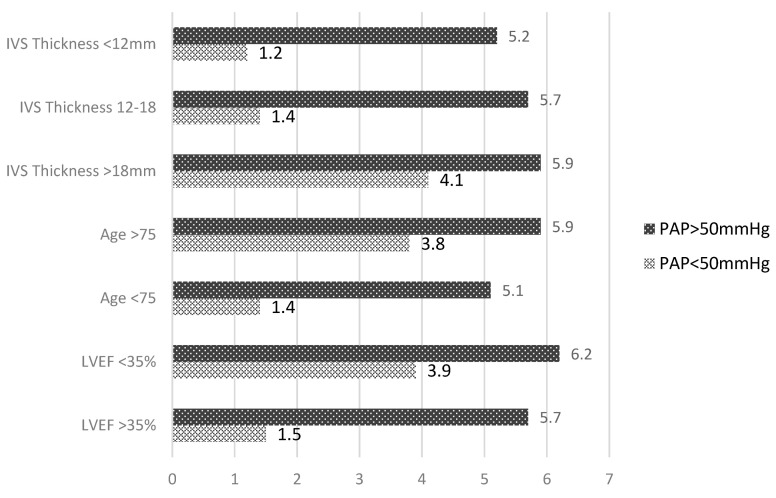
Early postoperative risk of death in patients with aortic stenosis depending on the mean PAP.

In Figure 4, the relative risks for death in the early postoperative period for the studied group are presented, associated with different parameters known to increase the mortality rates in AS patients having undergone surgery, such as: severe left ventricular hypertrophy, age, and left ventricular systolic performance. The relative risks are presented separately depending on the pulmonary artery pressure value.

The predictive value for death at 2 years postoperatively of left ventricular systolic dysfunction, age > 75 years, or interventricular septum thickness was the same in patients with PHT > 50 mmHg and higher in patients with normal PAP. In these patients, left ventricular ejection fraction < 35%, age > 75 years, or interventricular septum thickness >18 mm increased by 4-fold the risk of death at 2 years postoperatively.

The presence of PHT > 50 mmHg homogenized the relative early postoperative risk of death. It was increased regardless the left ventricular systolic performance, patients’ age, or the presence of the left ventricular hypertrophy.

**Figure 5:**
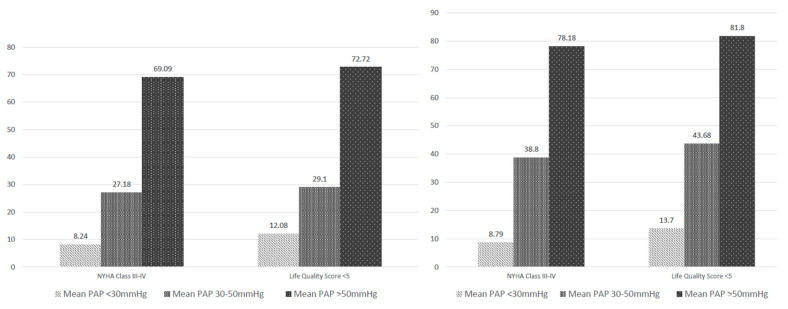
Patients’ evolution from the point of view of NYHA class and health-related quality of life score depending on the presence of pulmonary hypertension.

From a clinical point of view, the percent of patients with unfavorable evolution (NYHA class for heart failure > 3 and health-related quality of life score < 5) was higher in the subgroup with PHT > 50 mmHg regardless of the left ventricular systolic performance. In a multivariate analysis, the medium–high baseline PAP and presence of comorbidities (especially chronic obstructive pulmonary disease—COPD) were independent predictors of unfavorable evolution, but not the presence of LV systolic dysfunction (*p*-value = 0.000003, 95% CI = 9.8, respectively, 6.5). Thus, at 1-year follow-up, the percent of patients with NYHA class of heart failure 3 and 4 was almost 8-fold higher in patients with PHT > 50 mmHg in comparison with normal PAP, regardless of the systolic dysfunction (Figure 5).

At the same time, in both years of follow-up, we found a significant correlation between preoperative PAP and the quality of life. The health-related quality of life score < 5 was found in almost 6-fold more patients with PHT > 50 mmHg compared to patients with normal PAP (*p*-value = 0.00003, 95% CI = 5.8, respectively, 6.2).

The 2-year follow-up showed a significant correlation between PHT regression and the left ventricular systolic and diastolic performance as well as the preoperative dimensions of LV cavities (LV end-diastolic diameter, *p*-value = 0.02; LV end-systolic diameter *p*-value < 0.0001).

Other preoperative clinical and hemodynamical parameters or echographic parameters of the aortic prosthesis (LV-Ao gradient, prosthesis functional area) were not correlated with the PHT regression and, therefore, they cannot be used as predictors of its evolution.

## 4. Discussion

The main mechanism of PHT is represented by vascular proliferation and remodeling. It is a disease of small pulmonary arteries, leading to right ventricular heart failure and death. The presence of transpulmonary pressure gradient in patients with severe AS indicates the importance of an increase in pulmonary arterial resistance [10].

Additionally, an impressive increase in pulmonary arteriolar resistance was found in some patients with end-stage AS dying suddenly or deteriorating suddenly after catheterization. The rare finding of severe PHT in AS should be considered an important marker for sudden death; in association with LV, it may indicate an urgent need for valve replacement, regardless of the apparent clinical condition of the patient. PHT was also found to be associated with a higher mortality rate in patients with AS treated with trans-catheter aortic valve implantation (TAVI), while a decrease in PAP following intervention was a predictor for a better post-survival [11].

The prognostic significance of high PAP in patients with severe AS remains controversial [12]. Although our study is limited in evaluating only the patients with surgical AVR, it is one of the first large follow-ups analyzing the impact of PHT in patients with severe AS.

Unlike a previous cardiac-catheterization-based study which reported long-term mortality results [5], it showed that the postoperative mortality after AVR of the patients with AS and severe preoperative PHT was similar to those with mild-to-moderate preoperative PHT. Baseline PHT did not affect survival in these patients after AVR. This study, however, included a significantly smaller number of patients. Additionally, this study reported similar postoperative NYHA class III to IV and length of hospital stay in both mild-to-moderate and severe PHT groups, but the number of patients undergoing AVR was significantly smaller than in our study group.

Multivariate analysis, which was possible in our study thanks to the larger sample size, showed that the presence of medium–high baseline PAP and the comorbidities (especially COPD) predicted independently an unfavorable evolution. The preoperative LV systolic performance did not influence significantly the evolution of these patients at 2 years postoperatively. Additionally, similar to other previous studies, the postoperative reduction in the PAP value was one of the independent predictors of survival and higher health-related quality of life score after AVR [13].

Some studies have demonstrated a rapid reduction in systolic, diastolic and mean PAP after AVR, resulting in an improvement in clinical outcomes [14], but there are no studies evaluating the quality of life score and NYHA class at 2 years postoperatively after surgical AVR.

Most of the published papers measured mortality and the change in PAP in the immediate postoperative period, but because LV remodeling requires time after the surgery, the evaluation of medium-term outcomes provides more useful information.

Although all the patients in the study group had undergone surgical AVR, other studies suggested that there is better survival with AVR compared with medical management in these patients.

One observational study evaluated the risk factors for the development of PHT in patients with severe AS and investigated a possible protective effect of statin therapy against the development of PHT. Statins have pleiotropic effects which may be helpful in attenuating the progression of PHT because of their antiproliferative, antioxidant, and anti-inflammatory effects and endothelial-cell-function-maintaining properties [15,16,17]. It is advisable that more future research should focus on the efficacy and safety of statin therapy against PHT development, or for attenuating PHT progression.

On the other hand, mortality after AVR varies in different studies depending on the definition of severe PHT, the comorbidities and the study group structures (exclusion criteria), varying between 34% mortality at 5-year follow-up [18] and 32% mortality at a median of 460 days follow-up [19].

Additionally, in another study based on echocardiographic examination (comparing surgical AVR with only medical treatment in patients with severe AS and severe PHT), the long-term survival curves of patients with severe AS who underwent AVR were similar, irrespective of their PAP, but the number of patients was small [18].

Another study assessed the impact of LV systolic and diastolic performance on early and late outcomes in severe AS after AVR [20]. LV hypertrophy associated with chronically elevated LV systolic pressure and secondary myocardial fibrosis causes diastolic dysfunction in patients with AS, without having a well-defined clinical role in the management of these patients. After the replacement of the aortic valve, because of the remodeling of the LV, the diastolic dysfunction gradually improves, but not always completely. Severe baseline diastolic dysfunction is associated with increased mortality and adverse events, even after AVR [21].

In another study, LV-restrictive diastolic dysfunction and severe PHT were predictors of mortality in multivariate analysis, but LV systolic performance was not found as an independent predictor of mortality. However, perioperative mortality was independent of the severity of LV systolic dysfunction or concomitant coronary artery bypass grafting. AVR was associated with a significant improvement in LV ejection fraction, the severity of PHT and NYHA functional class. The difference between long-term survival of the operative survivors and the expected survival from life tables was not statistically significant in other studies.

Additionally, other studies have demonstrated a correlation between the presence of PHT and increased PAP and LV diastolic dysfunction in patients with severe AS [22].

The question remains regarding whether to operate or not on patients with severe AS and severe PHT, and what kind of intervention is optimal.

Although severe PHT was an independent predictor of perioperative mortality, it should not be a contraindication for AVR in patients with severe AS.

The present study is the first one which evaluates long-term prognosis in patients with AS with added PHT and the influence of PHT on postoperative course in these patients. Our study suggests that the assessment of the benefit for the individual patient is likely to consider the inherent risk of the type of the planned procedure. The impact of associated severe PHT for the decision to proceed with medication or with a conventional surgical or interventional procedure seems to be very important and it should be taken into consideration in further, larger, randomized studies.

Study limits. First, although this is one of the largest studies on echocardiography and diastolic filling in AS, the number of patients was still moderate. Second, the data on PAP and LVDFP were obtained from reports rather than cardiac catheterization (RHC). For this reason, it was not possible to analyze data from interventional assessment of LVDFP and RHC, thus making the analyzed group more heterogeneous. However, the C.C Iliescu Emergency Institute for Cardiovascular Diseases has a long tradition of evaluating patients with valve disease and all cardiologists performing these examinations are experienced in this regard. Third, the calculation of PAP based on trans-tricuspid gradient is subject to error; echocardiography cannot be reliable alone in the proper decision making of diagnosis and management of pulmonary arterial hypertension. It is a simple and non-invasive way for screening suspected patients, but the procedure for diagnosis and follow-up of the cases of PAP remains RHC [23]. Moreover, since PAP was determined by ultrasound measurements, we could not differentiate between fixed (defined as elevated pulmonary pressures with a transpulmonary gradient >15 mmHg, pulmonary vascular resistance (PVR) > 5 mmHg and PA diastolic-wedge gradient >7 mmHg, not reversible with pharmacological agents) and non-fixed PHT, with this requiring measurements obtained by RHC [24]. Additionally, all the patients in our study benefited from surgical AVR without having a comparison with a possible control group in which transcatheter implantation was performed.

## 5. Conclusions

1. The relevant independent predictors for PHT occurrence in surgical AS were: age > 75 years, restrictive LVDFP and the presence of moderate MR.
2. The presence of a severe PHT in patients with AS undergoing surgical AVR was associated with an early postoperative increased mortality rate. The mean PAP is a more reliable parameter for prognosis appreciation than the LV systolic function.
3. The independent predictors for increased early postoperative mortality rate in patients with surgical AS were:
  - Mean PAP > 50 mmHg;
  - The presence of a restrictive LVDFP;
  - Age > 75 years;
  - Interventricular septum thickness >18 mm;
  - Comorbidities (diabetes mellitus and pulmonary diseases).
4. PHT regression at 2 years postoperatively was only correlated with preoperative left ventricular dimensions and systolic and diastolic function and could not be predicted by other clinical or ultrasound parameters.

## Data Availability

All data generated or analyzed during this study are included in this published article.

## Appendix A

**Table A1:** Health-related quality of life questionnaire, adapted, abbreviated.

MOBILITY	0	Unable to control or use arms and legs.
	1	Requires help of another person or mechanical equipment (such as canes, crutches, braces, or wheelchair) to walk or get around independently.
	2	Able to walk, bend, lift, jump, and run normally for age
EMOTION	0	Almost always fretful, angry, irritable, anxious, or depressed.
	1	Occasionally or often fretful, angry, irritable, anxious, depressed, or suffering “night terrors”.
	2	Generally happy and free from worry.
SENSATION	0	Not able to see, hear, and speak normally for age.
	1	Sees, hears, or speaks with limitations or with equipment.
	2	Able to see, hear, and speak normally for age.
SELF-CARE	0	Requires the help of another person to eat, bathe, dress, or use the toilet.
	1	Requires help to eat, bathe, dress, or use the toilet independently.
PAIN	0	Frequent or severe pain; frequent disruption of normal activities. Discomfort requires prescription narcotics for relief
	1	Occasional pain. Discomfort relieved by non-prescription drugs or self-control activity without disruption of normal activities.
	2	Free or occasional pain and discomfort.
