# Correction: Iliuta et al. Impact of Pulmonary Hypertension on Mortality After Surgery for Aortic Stenosis. *Medicina* 2022, *58*, 1231

**DOI:** 10.3390/medicina62061111

**Published:** 2026-06-08

**Authors:** Luminita Iliuta, Marius Rac-Albu, Madalina-Elena Rac-Albu, Andreea Andronesi

**Affiliations:** 1 Department of Medical Informatics and Biostatistics, University of Medicine and Pharmacy “Carol Davila”, 050474 Bucharest, Romania; luminita.iliuta@umfcd.ro (L.I.); madalina.rac-albu@umfcd.ro (M.-E.R.-A.); 2 Cardioclass Clinic for Cardiovascular Disease, 031125 Bucharest, Romania; 3 Nephrology Department, University of Medicine and Pharmacy “Carol Davila”, 050474 Bucharest, Romania; 4 Nephrology Department, Fundeni Clinical Institute, 022328 Bucharest, Romania

## Text Correction

In the original publication [1], authors did not provide proper explanation of their statistical analyses, which required correction to explain that the RRs provided in the manuscript correspond to ORs. A correction has been made in the Abstract paragraph; the sixth sentence was revised as following: “The presence of severe PHT was independently associated with early postoperative mortality, with an odds ratio of 7.6, irrespective of other covariates.” Also, the eighth sentence of the abstract is also revised to “The presence of a severe PHT in patients with AS undergoing surgical AVR was associated with early postoperative mortality”.

A correction has been made to the third sentence of the third paragraph in the Introduction section: “Data regarding the specific influence of PHT on postoperative clinical course remain limited”.

A correction has been made in the second sentence of the first paragraph of the Material and Methods section “Patients with chronic atrial fibrillation, associated aortic valve insufficiency, acute myocardial infarction, or coronary lesions, associated mitral or tricuspid lesions were not included”. Corrected also were the fourth and fifth paragraph of the same section: “Categorical variables are presented as counts and percentages. Continuous variables are expressed as mean ± standard deviation (SD).

Odds ratios (ORs) with corresponding 95% confidence intervals were calculated using multivariable logistic regression models to assess:

1. The occurrence of PHT in patients with AS;
2. The association between PHT and cardiovascular complications as well as early postoperative mortality; and
3. Predictors of early postoperative mortality in patients with AS, including analyses stratified by mean pulmonary artery pressure (mPAP).

These analyses were conducted across three predefined patient subgroups. Multi-variate logistic regression models were used to estimate odds ratios (ORs) and corresponding 95% confidence intervals (CIs) for relevant covariates. A *p*-value < 0.05 was considered statistically significant. All statistical analyses were performed using SPSS software (version 20.0)”.

A correction has been made in the Results section: “Figure 1 presents the odds ratios for parameters associated with the occurrence of PHT in patients with severe AS. Age >75 years was associated with approximately 6-fold higher odds of PHT (OR = 6). A restrictive left ventricular diastolic filling pattern was also strongly associated with PHT occurrence. Specifically, an E/A velocity ratio >2 was associated with 12-fold higher odds (OR = 12), and an E-wave deceleration time <120 ms with 9-fold higher odds (OR = 9). Additionally, moderate mitral regurgitation was associated with approximately 9-fold higher odds of PHT occurrence. The presence of severe PHT was independently associated with approximately 9.8-fold higher odds of early postoperative death in multivariable analysis (OR = 9.8). Figure 2 illustrates the odds ratios for early postoperative mortality (at 5 and 10 days) and cardiovascular complications stratified according to mean PAP levels.

Multivariable logistic regression analysis was performed to assess factors associated with early postoperative mortality. Figure 3 presents the odds ratios for the variables included in the model. The following parameters remained independently associated with early postoperative mortality:

− Mean PAP > 50 mmHg (OR = 9.8, *p* < 0.001);
− Restrictive LV diastolic filling pattern (OR = 9.0, *p* < 0.001);
− Age > 75 years (OR = 6.8, *p* < 0.001);
− Interventricular septal thickness > 18 mm (OR = 4.2, *p* < 0.05);
− Presence of comorbidities (diabetes mellitus and chronic kidney disease) (OR = 6.5, *p* < 0.0001).

Parameters of left ventricular systolic function (LVEF), indices of aortic stenosis severity (transvalvular gradient and valve area), and left ventricular dimensions were not independently associated with early postoperative mortality. Additionally, a systolic-to-diastolic pulmonary venous flow ratio <1 (OR = 5.7), age >75 years (OR = 6.8), eccentric left ventricular hypertrophy with septal thickness >18 mm (OR = 4.2), and the presence of comorbidities were significantly associated with early postoperative mortality. Figure 4 illustrates postoperative mortality stratified by mean PAP levels in the study cohort. Differences in observed mortality were noted according to selected clinical and echocardiographic characteristics commonly associated with adverse out-comes in patients with AS undergoing surgery. Higher mortality rates were observed among patients with severe left ventricular hypertrophy, advanced age, and impaired left ventricular systolic function. Mortality values are presented separately according to PAP strata, allowing comparison across different degrees of PHT.

Differences in early postoperative mortality according to left ventricular systolic function, age, and interventricular septal thickness were less pronounced in patients with PHT >50 mmHg compared with those with normal PAP (Figure 4).

In the multivariable logistic regression model for early postoperative mortality (Figure 3), age >75 years and interventricular septal thickness >18 mm were independently associated with increased odds of death (OR = 6.8 and OR = 4.2, respectively), whereas left ventricular ejection fraction <35% showed a weaker association (OR = 1.9).

Among patients with PHT >50 mmHg, early postoperative mortality remained consistently high across subgroups defined by left ventricular systolic function, age, and left ventricular hypertrophy.

From a clinical perspective, the proportion of patients with unfavorable evolution (NYHA class III–IV and health-related quality of life score <5) was higher in the sub-group with mean PAP >50 mmHg, irrespective of left ventricular systolic function (Figure 5). In multivariable logistic regression analysis, higher baseline PAP and the presence of comorbidities (particularly chronic obstructive pulmonary disease) were independently associated with unfavorable clinical evolution, whereas left ventricular systolic dysfunction was not. At one-year follow-up, a higher proportion of patients with PAP >50 mmHg were in NYHA class III–IV compared with those with normal PAP (Figure 5).

At both one-year and two-year follow-up, higher preoperative PAP values were associated with poorer health-related quality of life. A greater proportion of patients with PAP >50 mmHg had a health-related quality of life score <5 compared with those with normal PAP (*p* = 0.00003).

At two-year follow-up, regression of PHT was significantly associated with left ventricular systolic and diastolic function, as well as with preoperative left ventricular cavity dimensions (LV end-diastolic diameter, *p* = 0.02; LV end-systolic diameter, *p* < 0.0001).

Other preoperative clinical and hemodynamic parameters, as well as echocardiographic parameters of the aortic prosthesis (LV–aortic gradient and prosthesis functional area), were not significantly associated with PHT regression and were not identified as independent predictors of its evolution.”

A correction has been made in the seventeenth paragraph of the Discussion section “The present study provides longitudinal data regarding the long-term prognosis of patients with severe AS and concomitant PHT, as well as the influence of PHT on postoperative evolution”.

## Figure Legend

The correction was made in the figure legend of Figures 1–4 and the correct legends appear as below. The authors state that the scientific conclusions are unaffected. This correction was approved by the Academic Editor. The correct Figure 1 legend appears as follows: “Figure 1. Odds ratios for predictors of PHT occurrence in patients with AS”. The correct Figure 2 legend appears as follows: “Figure 2. Odds ratios for early postoperative mortality (at 5 and 10 days) and cardiovascular complications stratified by mean PAP levels”. The correct Figure 3 legend appears as follows: “Figure 3. Odds ratios for early postoperative mortality in patients with aortic stenosis”.

The correct Figure 4 legend appears as follows: “Figure 4. Postoperative mortality stratified by mean PAP and selected clinical parameters (age, interventricular septal thickness, and left ventricular ejection fraction). Values represent observed mortality rates within each subgroup”.

The authors state that the scientific conclusions are unaffected. This correction was approved by the Academic Editor. The original publication has also been updated.
